# Supplementary material for: Multi‐dataset identification of innovative feature genes and molecular mechanisms in keratoconus
Source: J Cell Mol Med. 2024 Sep 19;28(18):e70079. doi: 10.1111/jcmm.70079 (PMC11412914; doi:10.1111/jcmm.70079)
Supplement: Supplementary file 2 — Material S2. [file JCMM-28-e70079-s002.zip › 7.GSEA analysis/1.ARL11/KEGG_NOTCH_SIGNALING_PATHWAY.html]

Details for gene set KEGG\_NOTCH\_SIGNALING\_PATHWAY[GSEA]

|  || Dataset | ARL11.group.cls#h\_versus\_l |
| Phenotype | group.cls#h\_versus\_l |
| Upregulated in class | l |
| GeneSet | KEGG\_NOTCH\_SIGNALING\_PATHWAY |
| Enrichment Score (ES) | -0.24694833 |
| Normalized Enrichment Score (NES) | -0.93051374 |
| Nominal p-value | 0.5206612 |
| FDR q-value | 1.0 |
| FWER p-Value | 1.0 |
Table: GSEA Results Summary

  

Fig 1: Enrichment plot: KEGG\_NOTCH\_SIGNALING\_PATHWAY      
 Profile of the Running ES Score & Positions of GeneSet Members on the Rank Ordered List

  

| SYMBOL | TITLE | RANK IN GENE LIST | RANK METRIC SCORE | RUNNING ES | CORE ENRICHMENT || 1 | NOTCH4 | na | 3905 | 0.204 | -0.0558 | No |
| 2 | MAML3 | na | 4364 | 0.188 | -0.0168 | No |
| 3 | HES5 | na | 4758 | 0.173 | 0.0202 | No |
| 4 | DTX3L | na | 6297 | 0.129 | 0.0119 | No |
| 5 | DTX1 | na | 6740 | 0.118 | 0.0319 | No |
| 6 | CREBBP | na | 7701 | 0.098 | 0.0315 | No |
| 7 | NOTCH3 | na | 8450 | 0.084 | 0.0333 | No |
| 8 | CTBP1 | na | 8519 | 0.082 | 0.0543 | No |
| 9 | NCSTN | na | 9705 | 0.064 | 0.0380 | No |
| 10 | KAT2A | na | 10034 | 0.060 | 0.0452 | No |
| 11 | NOTCH1 | na | 10709 | 0.051 | 0.0400 | No |
| 12 | LFNG | na | 10816 | 0.050 | 0.0508 | No |
| 13 | PTCRA | na | 12735 | 0.029 | 0.0037 | No |
| 14 | HDAC1 | na | 12805 | 0.029 | 0.0097 | No |
| 15 | JAG1 | na | 13628 | 0.022 | -0.0080 | No |
| 16 | PSEN2 | na | 14206 | 0.017 | -0.0198 | No |
| 17 | CIR1 | na | 14614 | 0.014 | -0.0276 | No |
| 18 | DTX2 | na | 14643 | 0.014 | -0.0245 | No |
| 19 | MAML2 | na | 14708 | 0.013 | -0.0226 | No |
| 20 | DLL4 | na | 14832 | 0.012 | -0.0227 | No |
| 21 | NCOR2 | na | 15400 | 0.008 | -0.0367 | No |
| 22 | JAG2 | na | 15563 | 0.007 | -0.0394 | No |
| 23 | MAML1 | na | 16649 | -0.001 | -0.0705 | No |
| 24 | PSEN1 | na | 16871 | -0.002 | -0.0762 | No |
| 25 | NOTCH2 | na | 17063 | -0.004 | -0.0807 | No |
| 26 | CTBP2 | na | 17070 | -0.004 | -0.0799 | No |
| 27 | APH1A | na | 17550 | -0.007 | -0.0917 | No |
| 28 | PSENEN | na | 18265 | -0.012 | -0.1089 | No |
| 29 | DVL3 | na | 19519 | -0.021 | -0.1393 | No |
| 30 | DTX3 | na | 20399 | -0.027 | -0.1572 | No |
| 31 | SNW1 | na | 21604 | -0.036 | -0.1819 | Yes |
| 32 | HDAC2 | na | 21663 | -0.037 | -0.1734 | Yes |
| 33 | DVL2 | na | 22249 | -0.042 | -0.1784 | Yes |
| 34 | MFNG | na | 22320 | -0.043 | -0.1684 | Yes |
| 35 | RFNG | na | 23095 | -0.051 | -0.1765 | Yes |
| 36 | HES1 | na | 23536 | -0.056 | -0.1735 | Yes |
| 37 | KAT2B | na | 23672 | -0.058 | -0.1612 | Yes |
| 38 | DVL1 | na | 23820 | -0.060 | -0.1487 | Yes |
| 39 | EP300 | na | 23862 | -0.060 | -0.1331 | Yes |
| 40 | RBPJ | na | 24118 | -0.064 | -0.1226 | Yes |
| 41 | DLL3 | na | 24209 | -0.065 | -0.1070 | Yes |
| 42 | ADAM17 | na | 24531 | -0.070 | -0.0968 | Yes |
| 43 | NUMB | na | 26419 | -0.097 | -0.1242 | Yes |
| 44 | DLL1 | na | 28356 | -0.140 | -0.1411 | Yes |
| 45 | NUMBL | na | 30771 | -0.213 | -0.1512 | Yes |
| 46 | DTX4 | na | 34092 | -0.434 | -0.1261 | Yes |
| 47 | RBPJL | na | 34460 | -0.518 | 0.0077 | Yes |
Table: GSEA details [plain text format]

  

Fig 2: KEGG\_NOTCH\_SIGNALING\_PATHWAY      
 Blue-Pink O' Gram in the Space of the Analyzed GeneSet

  

Fig 3: KEGG\_NOTCH\_SIGNALING\_PATHWAY: Random ES distribution      
 Gene set null distribution of ES for **KEGG\_NOTCH\_SIGNALING\_PATHWAY**
